# Greater Intermanual Transfer in the Elderly Suggests Age-Related Bilateral Motor Cortex Activation Is Compensatory

**DOI:** 10.1080/00222895.2014.981501

**Published:** 2015-01-09

**Authors:** Sara Graziadio, Kianoush Nazarpour, Sabine Gretenkord, Andrew Jackson, Janet A. Eyre

**Affiliations:** ^a^ Institute of Neuroscience, Newcastle University, England; ^b^ School of Electrical and Electronic Engineering, Newcastle University, England

**Keywords:** intermanual transfer, aging, HAROLD model, CRUNCH model

## Abstract

**ABSTRACT.** Hemispheric lateralization of movement control diminishes with age; whether this is compensatory or maladaptive is debated. The authors hypothesized that if compensatory, bilateral activation would lead to greater intermanual transfer in older subjects learning tasks that activate the cortex unilaterally in young adults. They studied 10 young and 14 older subjects, learning a unimanual visuomotor task comprising a feedforward phase, where there is unilateral cortical activation in young adults, and a feedback phase, which activates the cortex bilaterally in both age groups. Increased intermanual transfer was demonstrated in older subjects during feedforward learning, with no difference between groups during feedback learning. This finding is consistent with bilateral cortical activation being compensatory to maintain performance despite declining computational efficiency in neural networks.

Increased bilateral brain activation in older subjects during tasks where in the younger adult unilateral activation predominates, is a ubiquitous finding across a wide range of sensorimotor and cognitive tasks and has been demonstrated using a diverse range of techniques (e.g., Cabeza, 2002; Grady, 2013; Graziadio, Basu, Zappasodi, Tecchio, & Eyre, 2010; Hutchinson et al., 2002; Inuggi et al., 2011; Mattay et al., 2002; McGregor, Craggs, Benjamin, Crosson, & White, 2009; Naccarato et al., 2006; Sailer, Dichgans, & Gerloff, 2000; Ward & Frackowiak, 2003; Ward, Swayne, & Newton, 2008; Wu & Hallett, 2005; Yordanova, Kolev, Hohnsbein, & Falkenstein, 2004). The key unresolved issue is whether these age-related increases in bilateral brain activity are primarily adaptive or maladaptive. On one hand it has been proposed that this reflects additional recruitment of functioning neural networks to compensate for declining computational efficiency with age, thereby maintaining performance levels—the Hemispheric Asymmetry Reduction in Older Adults (HAROLD) model (Cabeza, 2002) or delaying and slowing the rate of age related decline—the Compensation-Related Utilization of Neural Circuits Hypothesis (CRUNCH) proposal (Reuter-Lorenz & Cappell, 2008). The alternative hypothesis is that the increased areas of activation are a consequence of progressive dedifferentiation of the neural networks and an inability to suppress inappropriate processing, leading to conflict or reduced availability of appropriate resources and therefore causally related to decreases in performance with age (Bernard & Seidler, 2012; Lindenberger & Baltes, 1997; Zarahn, Rakitin, Abela, Flynn, & Stern, 2007). Resolving the issue of whether increased areas of activation are compensatory or maladaptive is of more than academic interest, as the two hypotheses lead to conflicting therapeutic approaches for rehabilitation. The former would imply increasing the excitability of the ipsilateral motor cortex to augment performance whereas the later would indicate suppression of activity in the ipsilateral cortex as part of the rehabilitation strategy.

Despite the continued debate (Grady, 2013), there is still no consensus on which hypothesis is correct. This may be because overrecruitment of brain activity can be interpreted as compensatory when there is a positive correlation between cortical activity and behavior (McIntosh et al., 1999), when this correlation is negative (Vincent, Kahn, Snyder, Raichle, & Buckner 2008) and even when performance in older adults is impaired (Zarahn et al., 2007), using the argument that the performance might have been even worse without the overrecruitment. We propose that distinguishing between these hypotheses may become clearer in situations where bilateral activation of neural networks during one task potentially conveys an advantage in performance of another function. Superior performance of this other function in older adults would indicate bilateral cortical activation recruits functioning neural networks relevant to the task, thereby providing strong evidence in support of compensatory mechanisms rather than being a consequence of an inability to suppress inappropriate neural processing.

We propose that intermanual transfer of motor skills to the opposite hand, while learning to perform a novel unimanual task, provides one such opportunity to look for superior performance in the elderly. When motor learning is accomplished with one limb, the ability to perform the same task with the untrained limb can also improve (Pereira, Raja, & Gangavalli, 2011; Perez, Wise, Willingham, & Cohen, 2007; Shea, Kovacs, & Panzer, 2011). This phenomenon is best illustrated by skilled movements such as writing, which are normally learnt by and performed only with the dominant arm and hand, however, when required to use the nondominant upper limb, for example when recovering from a broken arm, people show a reasonable degree of competence in writing with their nondominant hand. If increased bilateral activation of neural networks in the elderly is compensatory, then the resultant interhemispheric cooperation between functioning neural networks that share control of the performance of both hands, will lead to increased intermanual transfer of skill in the elderly compared to young adults.

We chose therefore to investigate intermanual transfer to the left hand of the motor skills acquired while young and older adults learn to perform with their right hand a novel myoelectric-controlled interface task comprising two sequentially linked action components (Graziadio et al., 2014; Nazarpour, Barnard, & Jackson, 2012; Radhakrishnan, Baker, & Jackson, 2008). The first component requires feedforward motor learning, when predominantly unilateral activation of motor networks in the young adults and bilateral activation in the elderly has been demonstrated across a range of visuomotor tasks involving hand muscles and using a range of experimental techniques (Mattay et al., 2002; Sailer et al., 2000; Seidler, Noll, & Thiers, 2004; Ward & Frackowiak, 2003; Wu & Hallett, 2005; Yordanova et al., 2004); the second component requires feedback learning, when bilateral activation of motor networks in both age groups has been consistently demonstrated (Grafton, Schmitt, Van Horn, & Diedrichsen, 2008; Noble, Eng, Kokotilo, & Boyd, 2011; Ullsperger, Harsay, Wessel, & Ridderinkhof, 2010). Our hypothesis was that the elderly subjects would have significantly greater intermanual transfer of motor learning for the first component but not for the second, where intermanual transfer of the motor skill would be similar to or less than that of the young adults.

## Methods

The study was approved by the ethical committee of Newcastle University and written informed consent was obtained from participants. All data used in this study is available freely in CARMEN data sharing platform (https://portal.carmen.org.uk/) under a folder created by Kianoush Nazarpour. Data files are visible to all people who have subscribed in the CARMEN platform. To comply with the requirements of our ethics approval, we have anonymized the data files; name and age information have been removed. However, subject groups, younger and older, are still identifiable. Note that, data from Sub 3 and Sub 10 in the old group have been removed from the analysis.

### Participants

In total, 24 right-handed subjects, with no history of a neurological disorder participated in this study: the younger group comprised 10 subjects, age 25–35 years old (28 ± 2 years). The older group comprised 14 subjects age 55–82 years old (67 ± 9 years). All participants were naive to the experimental setup and objectives.

### Experimental Setup

**FIGURE 1.**
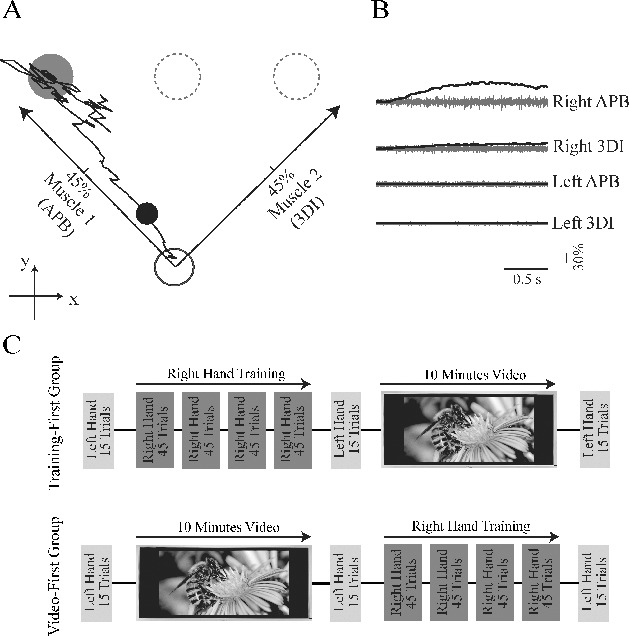
Task design. (**A**) Muscle pairs controlled the cursor along axes diagonal to screen axis. The cursor (the black circle) is at 120 ms from movement onset (corresponding to the distance index). (**B**) Control signals (darker traces) were computed at 75 Hz by rectifying and smoothing the preceding 500 ms of electromyogram signal (EMG; lighter traces) to determine the instantaneous position of the myoelectric cursor. (**C**) Experimental design, showing sequence of left hand testing blocks interspersed with right hand training and video presentation.

A MATLAB (MathWorks, Inc., Natick, MA) R14-based graphical user interface linked to Cogent2000 (Cogent 2000)^1^ was developed to implement these experiments. All subjects controlled a circular cursor with the electromyogram (EMG) signals recorded from abductor pollicis brevis and third dorsal interosseus muscles of either the left or right hand (Figure 1A). The cursor and the target were displayed on a computer screen positioned to allow the subjects to view both the target and the cursor position in a real-time display. The target diameter was > 16 mm, as Seidler et al. (2004) demonstrated that young adult subjects acquire a target of this size in a single aiming movement, relying on feedforward control, and there is unilateral hemispheric activation on functional magnetic resonance imaging (fMRI).

Subjects sat with their arms supported, flexed at the elbow with the forearm pronated. Isometric contraction of the hand muscles was achieved by squeezing against the resistance of a semicompliant object (a rugby ball). The object was attached firmly to the arm support so that the arm posture (position and orientation) was fixed for each subject during the experiment. The distance between the object and the subjects could be adjusted depending in the arm length. Surface EMGs from hand muscles were recorded using Ag/AgCl electrodes and high-pass filtered at 30Hz (Neurolog NL824, Digitimer, Welwyn Garden City, UK) before sampling at 5 KHz (NI USB-6259, National Instruments, Austin, TX) and stored on computer for offline processing.

### Myoelectric-Controlled Interface Task

The task comprised two actions. First, moving a cursor from a home base to a target positioned pseudorandomly in one of three positions and second, holding the cursor as steadily as possible within the target for 1 s. A full description is presented elsewhere (Nazarpour et al., 2012). At the start of the experiment, subjects were informed of the general task design and shown the hand movements that recruited each recorded muscle. They were then instructed to contract each muscle at a level. We ensured the compatibility between the EMGs on both hands empirically: At the beginning of the experiment we asked the subjects to squeeze both balls simultaneously at a level that could be comfortably maintained without fatigue, which in previous studies corresponded to between 10% and 20% of maximum voluntary contraction (Nazarpour et al. 2012; Radhakrishnan et al., 2008). We calibrated of the compatibility of the EMG signals by changing the amplifier gains and estimating the α s in Equation 1. The experimenter's screen, but not the subject's, showed four vertical bars. The heights of these bars corresponded to the normalized EMG activity level. Note that despite in training and testing blocks only two muscles were used, the calibration was done on all four muscles at the beginning of the experiment. Finally, to check whether they can repeat the same contraction, they were instructed to contract each muscle independently. If the vertical bar corresponding to each muscle did not significantly undershoot or overshoot the normalized value, the experiment began. Otherwise the above procedure repeated.

Cursor control signals were computed at 75 Hz (at 13-ms intervals) by smoothing (with a rectangular window) the preceding 500 ms of rectified EMG. The control signals from the pair of muscles determined the instantaneous position of the cursor along orthogonal axes 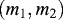 that were diagonal to screen coordinates 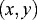 (Figure 1A):(1) 
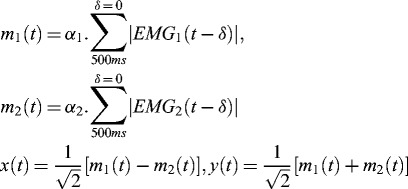
where, 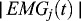 denotes the rectified activity of muscle *j* at time *t*. The coefficients 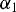 and 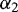 normalize the control signals by the comfortable contraction level.

The smoothing procedure slows the movement of the cursor. However, because the position of the cursor is updated every 13 ms, any change in the EMG amplitude takes effect instantaneously. In postexperiment conversation with the participants, we confirmed that the subjects did not notice or complain about cursor delays.

Subjects initiated a trial by relaxing their hand to bring the cursor to a central home zone; after 250 ms a target appeared. Targets were presented pseudorandomly at one of three locations, namely to the right, to the left, or centrally above the home position. The central target required 45% of comfortable contraction in both muscles (Figure 1A). Subjects were instructed to move the cursor towards the target as quickly as possible (aim period) and then to hold the cursor within the target for 1s (hold period; Figure 1B). At the end of each trial subjects were shown a score based on the distance between the cursor and the center of the target during the hold phase of the task and thus reflecting error correction. The score in each trial was computed with(2) 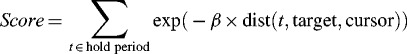
where 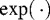 denotes the exponential function, 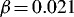 is a constant, and 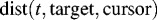 is the Euclidean distance between target and cursor at each time instance *t* during the hold period.

### Experimental Design

The experiment comprised five blocks (Figure 1C). Blocks 1, 3, and 5 comprised 16 trials during which the subjects controlled the cursor by contraction of the muscles of the left hand. In each age group, half of the subjects were randomly assigned to control the cursor with the muscles of the right hand in Block 2 (training session) and to watch a video in Block 4 (video session). The other half undertook the reverse order of video session (Block 2) followed by the right hand training (Block 4). The durations of the video and the training sessions were the same. The video session was introduced to control for the possibility that apparent intermanual transfer in fact represents consolidation of left hand learning following the initial short, baseline assessment of the naïve left hand performance. The right hand training session (either Block 2 or Block 4) comprised four subblocks, each of 45 trials giving a total of 180 training trials, and was used to estimate learning rate. The blocks pre- and posttraining (Blocks 1 and 3 or Blocks 3 and 5, depending on the session order) were used to estimate transfer of learning to the left hand after training of the right hand.

### Mirror Movements

To determine if there were significant differences between age groups in the probability of mirror movements in the left hand during right hand training (either Block 2 or Block 4), the rectified EMG of the left hand muscle was averaged across all trials for every subblock during the aim and the hold periods. A repeated measures analysis of variance (ANOVA) was used with within-subject factor period (aiming, holding) and a between-subject factor of age. There was no age effect for mirroring during either the aim or the hold phases of right hand training, *F*(1, 20) = 0.762, *p =* .394, η^2^_p_ = .039, indicating that, if mirroring occurred, it occurred with an equal probability across the age groups. There was a period effect, *F*(1, 20) = 9.267, *p =* .007, η^2^_p_ = .328, with higher mirroring during the holding than during the aiming period (0.056 ± 0.016 vs. 0.046 ± 0.014). There was no age by period interaction, *F*(1, 20) = 0.973, *p =* .336, η^2^_p_ = .049.

### Performance

Performance for both the trained right hand and the untrained left hand were estimated as follows:

*Feedforward component:* Performance was estimated from the center-to-center Euclidean distance between the cursor and the target during the first aiming movement at 120 ms after the reaction time, defined as crossing out of the starting zone indicated by the black circle.
*Feedback component:* Performance was estimated using the score displayed to the subject during the task at the end of each trial, which reflects feedback correction of errors during the hold period.

Note, for distance lower scores indicate a higher performance while for *score* higher values indicate higher performance levels. The two performance measures were averaged across all trials within a block for the left hand (16 trials) or a subblock for the right hand (45 trials).

### Intermanual Transfer and Rate of Learning

Indexes for intermanual transfer (Equation 3) and rate of learning (Equation 4) were computed as:(3) 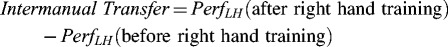

(4) 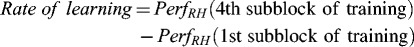



To ensure that a positive value indicated learning and intermanual transfer for both *score* and *distance*, the indexes for *distance* were multiplied by −1.

### Statistical Analysis

The data were normally distributed as demonstrated by the not significant Kolmogorov-Smirnov test (*p* > .15). Significance was set at *p* < .05, with Bonferroni correction. A general linear model ANOVA was used with Greenhouse-Geisser correction if required (SPSS 15, SPSS Inc, Chicago, IL, USA). Residuals versus fitted values, normality of the residuals, influential points, and outliners were investigated before accepting a model.

#### Effect of order of presentation of the training and video blocks

A between-subject factor order (right hand training first, video first) was used in a multivariate ANOVA design. There was no order effect for intermanual transfer and rate of learning for both score and distance, *F*(4, 17) = 0.698, *p =* .644, η^2^_p_ = .032. The two groups (right hand training first, video first) were therefore collapsed together for all subsequent analyses.

#### Age group differences

To investigate age differences in the overall performance of the task a repeated measures ANOVA was used for score and distance separately: within-subject factor of side (Right and Left) and subblocks (for right hand: four subblocks; for left hand: before/after video, before/after right hand training) and a between-subjects effect of age (young, older) were included.

To investigate the effect of age on rate of learning, the full model was reduced by side for both the score and the distance indexes and the right hand performances were studied. For the left hand assessments, the before/after right hand training and the before/after video performances were analyzed separately (within-subject factor with two levels sequence) with the same repeated measure design.

To investigate the effect of age or rate of learning on intermanual transfer a univariate ANOVA was used with age and rate of learning as between-subject factor.

## Results

In two old subjects the 50 Hz noise level increased substantially during the course of the experiment. Although we adjusted for it by recalibrating the EMG gains, this inevitably meant the cursor behavior changed. Since recalibration this is likely to have interrupted the ongoing learning of the motor program these subjects were excluded from the analysis.

### Performance of Task

**FIGURE 2.**
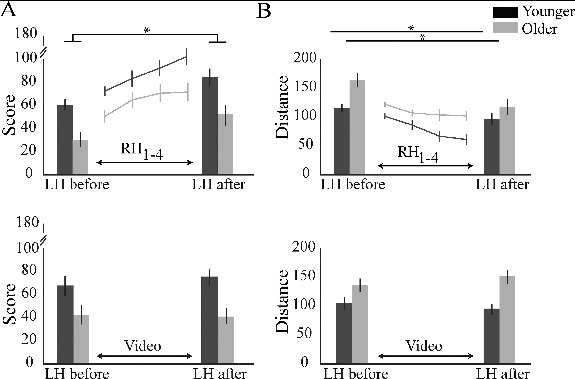
Performance using score and distance indexes. (**A** and **B**, top) Bar graph indicates performance of the left hand (LH) before and after right hand (RH) training; line graph indicates RH performance across the four training subblocks averaged across all target positions. (**A** and **B**, bottom) Bar graph indicates averaged performance of the LH before and after the subjects watched a video. Error bars represent standard error. *p <0.05.

An age effect was present across left hand and right hand performances for both score and distance indexes: score, *F*(1, 20) = 14.786, *p =* .002, η^2^_p_ = .401; distance, *F*(1, 20) = 10.282, *p =* .004, η^2^_p_ = .340, with the younger having significantly better performances than the older for both (Figure 2).

### Effect of Right Hand Training on Performance

#### Right hand performance

There was a significant effect of subblock on the right hand performance: score, *F*(1.98, 39.639) = 30.287, *p <* .001, η^2^_p_ = .602; distance: *F*(2.11, 42.2) = 12.833, *p <* .001, η^2^_p_ = .391 (Figure 2); there was no interaction with age (*p >* .2 for both score and distance).

#### Left hand performance

There was a significant effect of sequence for left hand performance with an important effect size, showing improved left hand performance after right hand training: score, *F*(1, 21) = 36.757, *p <* .001, η^2^_p_ = .648; distance, *F*(1, 21) = 30.966, *p <* .001, η^2^_p_ = .608 (Figure 2). There was no age by sequence interaction for score, *F*(1, 20) = 0.002, *p =* .966, η^2^_p_ = 0. There was a significant age by sequence interaction for distance, *F*(1, 20) = 5.368, *p =* .031, η^2^_p_ = .212. We investigated this interaction reducing the model by age the improvement in performance for the left hand after right hand training was a trend in the younger, *F*(1, 9) = 4.518, *p =* .062, η^2^_p_ = .334), but highly significant in the older with a high effect size, *F*(1, 11) = 36.240, *p <* .001, η^2^_p_ = .767. There was no effect of sequence for video for both indexes: score, *F*(1, 20) = 0.405, *p =* .532, η^2^_p_ = .020; distance, *F*(1, 20) = 0.044, *p =* .836, η^2^_p_ = .002 (Figure 2).

### Rate of Learning Index

**FIGURE 3.**
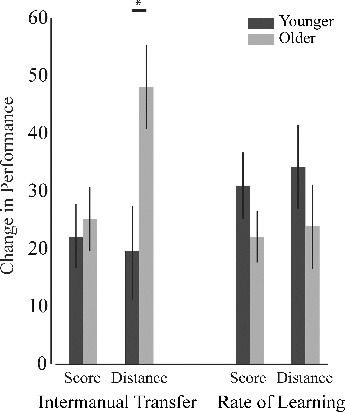
Intermanual transfer and rate of learning analysis. Intermanual transfer was greater in the older group for distance despite their comparable rate of learning. Error bars represent standard error. *p <0.05.

There was no effect of age for the rate of learning index for score, *F*(1, 20) = 1.123, *p =* .302, η^2^_p_ = .064, or for distance, *F*(1, 20) = 0.868, *p =* .363, η^2^_p_ = .056 (Figure 3).

### Intermanual Transfer Index

There was no effect of age for the intermanual transfer index for score, *F*(2, 21) = 0.320, *p =* .578, η^2^_p_ = .017, but there was a significant effect of age for the intermanual transfer index for the distance with a medium effect size, *F*(2, 21) = 7.185, *p =* .015, η^2^_p_ = .274), which was significantly higher in the older group (Figure 3) compared to the younger group. Only in the score index a significant effect of rate of learning on the intermanual transfer was observed, *F*(2, 21) = 7.063, *p =* .016, η^2^_p_ = .271.

## Discussion

Our findings confirm previous observations of preserved inter-manual transfer in older subjects (Hinder, Carroll, & Summers, 2013; Langan & Seidler, 2011; Lazarus & Haynes, 1997; Pan & Van Gemmert, 2013; Panzer, Gruetzmacher, Fries, Krueger, & Shea, 2011; Seidler, 2007; Wang, Przybyla, Wuebbenhorst, Haaland, & Sainburg, 2011). However, there have been some contradictory findings in the elderly. While performance gains in ballistic abductions of the right index finger, were transferred to the left hand in young adults, they were not in the elderly (Hinder, Schmidt, Garry, Carroll, & Summers, 2011), implying an absence of interlimb transfer. Using the same ballistic task, however, Parikh and Cole (2013) later demonstrated preserved intermanual transfer although it was reduced in comparison to younger adults. In that study the same older subjects demonstrated no impairment of inter-manual transfer of a more complex grip and lift task compared to younger adults. The ballistic finger abduction task is a simple task that requires movements about a single joint performed as fast as possible. In contrast studies demonstrating preserved intermanual transfer in the elderly have used more complex, usually visuomotor tasks. It may be possible that the interlimb transfer observed with these more complex tasks reflects in part transfer of a cognitive component. Another difference between ballistic and visuomotor tasks is the role of proprioceptive feedback for learning. Focusing on proprioception increases motor learning of a ballistic task (Rosenkranz & Rothwell, 2012), while perturbation of proprioception does not affect visuomotor learning (Pipereit, Bock, & Vercher, 2006). Since proprioception deteriorates during aging (Goble, Coxon, Wenderoth, Van Impe, & Swinnen, 2009) the difference in the proprioceptive load when learning tasks is likely to confound studies of interlimb transfer.

The hypothesis for our study, however, did not center on age-related changes in intermanual transfer per se, but rather to use it as a paradigm to consider if bilateral brain activation during a task reflected compensatory mechanisms (Cabeza, 2002; McIntosh et al., 1999; Reuter-Lorenz & Cappell, 2008; Ward, 2006) or was maladaptive as a consequence of age related dedifferentiation of the neural networks (Bernard & Seidler, 2012; Lindenberger & Baltes, 1997; Zarahn et al., 2007).

### Increased Intermanual Transfer of Feedforward Components in Older Subjects

Motor behaviors occur along a continuum of control, ranging from feedforward to feedback mechanisms and we studied these processes with two indexes, the score and the distance, respectively. The score index, is a performance metric that indicates the subject's ability to correct for errors interactively during the 1s hold period, using available feedback. Feedback mechanisms are bilateral in both younger and older subjects (Grafton et al., 2008; Noble et al., 2011; Ullsperger et al., 2010). To support this proposal the rostral cingulate zone and the anterior insula are two examples of brain areas bilaterally involved in performance monitoring and error correction that are similarly activated in older subjects and in younger subjects during steady, low level isometric contractions of either hand (Noble et al., 2011; Ullsperger et al., 2010; Ward et al., 2008). Our finding of no difference in the intermanual transfer for the score index between younger and older subjects is what would be predicted from these functional imaging studies, if both age groups activated bilateral functional neural networks that are shared by both hands in the performance of the task.

The distance index is based on the speed and accuracy of the feedforward execution of movement in the first 120 ms after movement onset, when there is little opportunity to correct errors based on feedback (Seidler et al., 2004). Feedforward control requires a predictive internal model of the limb and environment, which can subsequently be updated based on sensory feedback (Shadmehr, Smith, & Krakauer, 2010). The learning process tends to reduce the discrepancies between expectation and actual experience, updating the internal model and reducing the prediction error (Mehta & Schaal, 2002; Shadmehr et al., 2010). Repeated studies have demonstrated a difference in brain activation patterns between young and older subjects during feedforward visuomotor tasks, with predominantly unilateral activation in the young and bilateral activation in the elderly (Mattay et al., 2002; Sailer et al., 2000; Seidler et al., 2004; Ward & Frackowiak, 2003; Wu & Hallett, 2005; Yordanova et al., 2004). Our finding of a significantly increased intermanual transfer for the distance index in the elderly, despite an overall lower performance level, is consistent with the proposal that age related bilateral activation involves recruiting functioning neural networks that are shared by either hand during performed of the task, thus facilitating transfer of skill between hands.

We considered, however, whether a ceiling effect from saturation of right hand performance during training in the younger adults, led to an underestimate of their intermanual transfer. We were able to exclude this explanation, since the rate of learning for the distance index during right hand training continued across the four subblocks for both age groups (Figure 3) and there was no difference in the rate of learning between younger and older in the last subblock of right hand training (calculated as the difference between the performance in the first 10 trails and last 10 trials in subblock 4; *p =* .565).

Another possible explanation is that the younger adults had a higher rate of learning with their left hand during the assessment of naive function (i.e., before right hand training), leading to a higher naive left hand performance and therefore an underestimate of total intermanual transfer. This is also an unlikely explanation because, although there was a difference between age groups in the rate of learning of the distance index for the left hand before training (calculated as the difference between the performance in the first four trails and last four trials), *F*(1, 20) = 7.407, *p =* .013, this arose because the young adults had a lower rate of learning in the left hand than the older adults (10 ± 10 vs. 48 ± 9, calculated in Equation 4). Furthermore the rate of learning with the left hand was not correlated with intermanual transfer (Spearman correlation, two-tails, *p =* .782).

### Conclusion

Our finding of increased intermanual transfer in older adults of feedforward motor learning is supported by the study of Wang et al. (2011) who examined the effect of aging on lateralization of feedforward intermanual transfer, similarly using a novel visuomotor task. Wang et al. found asymmetrical interlimb transfer of feedforward learning in young adults, with significant intermanual transfer only occurring from the nondominant to dominant hands and attributed it to the known lateralization for motor control during feed forward movement in young adults. In contrast symmetrical intermanual transfer was demonstrated in the elderly, which they concluded supports diminished hemispheric lateralization of feedforward motor control. Our study supports their findings and extends these observations by demonstrating significantly increased intermanual transfer in the elderly, but only of feedforward motor learning, and despite a lower overall proficiency in performing the task with their trained hand.

Our findings of increased intermanual transfer provides support for the hypothesis that age related bilateral activation recruits functioning neural networks involved in hand control and is therefore predominantly a compensatory mechanism (Cabeza, 2002; McIntosh et al., 1999; Reuter-Lorenz & Cappell, 2008; Ward, 2006), rather than being maladaptive, reflecting an inability to focus neural activity appropriately (Bernard & Seidler, 2012; Lindenberger & Baltes, 1997; Zarahn et al., 2007). These conclusions would have been strengthened by direct measurement of brain activation (electroencephalography [EEG], fMRI) or of cortical excitability of the contralateral or ipsilateral hemisphere using transcranial magnetic stimulation (TMS) during our studies. However, there are limits to the complexity of studies that elderly subjects can tolerate.

Recent evidence suggests that myoelectric-controlled interfaces can be an effective tool for retraining muscle activation patterns after stroke (Wright, Rymer, & Slutzky, 2013). Our results suggest that motor training or other therapies designed to increase the excitability of motor networks controlling the unaffected limb may exploit enhanced transfer and aid motor rehabilitation in the elderly following stroke.

## NOTE

1. This experiment was realized using Cogent 2000 developed by the Cogent 2000 team at the FIL and the ICN and Cogent Graphics developed by John Romaya at the LON at the Wellcome Department of Imaging Neuroscience.
